# Inferring Disease-Associated Microbes Based on Multi-Data Integration and Network Consistency Projection

**DOI:** 10.3389/fbioe.2020.00831

**Published:** 2020-08-04

**Authors:** Yongxian Fan, Meijun Chen, Qingqi Zhu, Wanru Wang

**Affiliations:** School of Computer Science and Information Security, Guilin University of Electronic Technology, Guilin, China

**Keywords:** disease, microbe, association prediction, multi-data integration, network consistency projection

## Abstract

Plenty of microbes in our human body play a vital role in the process of cell physiology. In recent years, there is accumulating evidence indicating that microbes are closely related to many complex human diseases. In-depth investigation of disease-associated microbes can contribute to understanding the pathogenesis of diseases and thus provide novel strategies for the treatment, diagnosis, and prevention of diseases. To date, many computational models have been proposed for predicting microbe–disease associations using available similarity networks. However, these similarity networks are not effectively fused. In this study, we proposed a novel computational model based on multi-data integration and network consistency projection for Human Microbe–Disease Associations Prediction (HMDA-Pred), which fuses multiple similarity networks by a linear network fusion method. HMDA-Pred yielded AUC values of 0.9589 and 0.9361 ± 0.0037 in the experiments of leave-one-out cross validation (LOOCV) and 5-fold cross validation (5-fold CV), respectively. Furthermore, in case studies, 10, 8, and 10 out of the top 10 predicted microbes of asthma, colon cancer, and inflammatory bowel disease were confirmed by the literatures, respectively.

## Introduction

As far as we know, microbes are ubiquitous in our living environment, and they occupy nearly all habitats including humans and animals (Kouzuma et al., 2015). According to existing literatures, the microbes are mainly classified into fungi, archaea, bacteria, protozoa, and viruses in the human body (Methé et al., 2012; Sommer and Bäckhed, 2013). More and more studies have shown that most of these microbes are friendly to human beings and play a significant role in the physiology processes of the human body, such as regulating gastrointestinal development, providing protection for pathogens, and enhancing metabolic capability (Ventura et al., 2009). Specifically, the overwhelming majority of microbes inhabit the gastrointestinal tract in an adult gut, where they not only synthesize essential vitamins and amino acids but also promote the digestion of indigestible components in the human diet (Huang et al., 2017). Thus, abnormal changes in the microbe communities may affect human health and diseases. For example, low microbial diversity could result in inflammatory bowel disease and obesity (Turnbaugh et al., 2009; Qin et al., 2010). However, high microbial diversity is associated with bacterial vaginosis in the vagina (Fredricks et al., 2005). Researchers have confirmed the close relationship between microbes and diseases. Some microbes may cause various diseases, such as colon cancer (Sears and Garrett, 2014), kidney stones (Hoppe et al., 2011), asthma (Hilty et al., 2010), colorectal carcinoma (Sobhani et al., 2011; Kostic et al., 2012), and inflammatory bowel disease (Frank et al., 2007). On the one hand, uncovering the disease-associated microbes can contribute to better understanding the pathogenesis of the diseases. On the other hand, understanding the mechanism of microbes behind the diseases provides novel strategies for the prevention, diagnosis, and treatment of the diseases (Zou et al., 2017; Peng et al., 2018). Unfortunately, the traditional biological experiments to uncover the relationship between microbes and diseases are time-consuming and costly. Thus, there is an urgent need to construct computational models to predict the disease-associated microbes.

In recent years, researchers have developed a number of feasible and effective prediction models for microbe–disease associations, which could provide the most promising disease-associated microbes for experimental verification. For example, according to the hypothesis that functionally similar microbes tend to be associated with similar diseases (Chen et al., 2016), Chen et al. (2016) proposed using the KATZ measurement to predict human microbe–disease associations (KATZHMDA) on a large scale. Huang et al. (2017) applied the designed depth-first search algorithm on the heterogeneous networks and proposed a path-based approach (PBHMDA) to reveal the microbes that are likely to be associated with the disease. Wang et al. (2017) developed a machine learning-based computational approach called LRLSHMDA, which calculates the association scores for microbe–disease pairs based on the known microbe–disease association network. Huang et al. (2017) developed a novel computational method (NGRHMDA), which can predict microbe–disease associations by applying collaborative recommendation model on a graph. Bao et al. (2017) proposed the computational model named NCPHMDA, which combines space consistency projection scores for diseases and microbes to predict latent disease-associated microbes. Zou et al. (2017) put forward a new prediction model called BiRWHMDA, which simultaneously performs random walks on the microbe similar network and disease similar network to uncover potential microbe–disease associations. Shi et al. (2018) proposed a predictive method based on Binary Matrix Completion (BMCMDA) for inferring the associations of microbe–disease.

However, the abovementioned methods have their own various shortcomings in uncovering microbe–disease associations. Multiple available similarity networks can be used for predicting disease–microbe associations. However, most of the previous methods are performed on individual networks, ignoring the complementarity between different similarity networks. How to better fuse them is still worth investigating. In this paper, to resolve the abovementioned limitations, we presented a novel computational model of multi-data integration and network consistency projection for prediction of Human Microbe–Disease Associations (HMDA-Pred) to boost the performance of human microbe–disease association prediction, which integrates multiple similarity networks. To begin with, the Gaussian interaction profile kernel similarity network and cosine similarity network for microbes and diseases were constructed based on known microbe–disease associations. Subsequently, we integrated the Gaussian interaction profile kernel similarity network of microbes and cosine similarity network of microbes by a linear network fusion method. In the same way, we integrated the Gaussian interaction profile kernel similarity network of diseases and cosine similarity network of diseases. Finally, we applied the network consistency projection algorithm to uncover the microbe–disease associations. Two evaluation strategies were implemented to evaluate the performance of HMDA-Pred, including leave-one-out cross validation (LOOCV) and 5-fold cross validation (5-fold CV). Related data and source code are available online at: https://github.com/AugustMe/HMDA-Pred.

## Materials and Methods

### Known Microbe–Disease Associations

We used the same microbe–disease associations as the existing literatures (Chen et al., 2016; Huang et al., 2017; Peng et al., 2018). The dataset was initially derived from the Human Microbe–Disease Association Database named HMDAD (Ma et al., 2016)^1^, which collected 483 microbe–disease associations from literatures. After removing duplicate associations of the dataset, we obtained 450 unique associations between 292 microbes and 39 diseases. Then, we constructed an adjacency matrix *MD*(*nm* × *nd*) to describe the association relationship between microbes and diseases, where *nm* and *nd* represented the number of microbes and diseases, respectively. If microbe *m*(*i*) was proved to be associated with disease *d*(*j*), the value of *MD*(*i*, *j*) was 1, otherwise 0. If the value of *MD*(*i*, *j*) is 0, that means there is no evidence yet showing microbe *m*(*i*) is associated with disease *d*(*j*).

**FIGURE 1:**
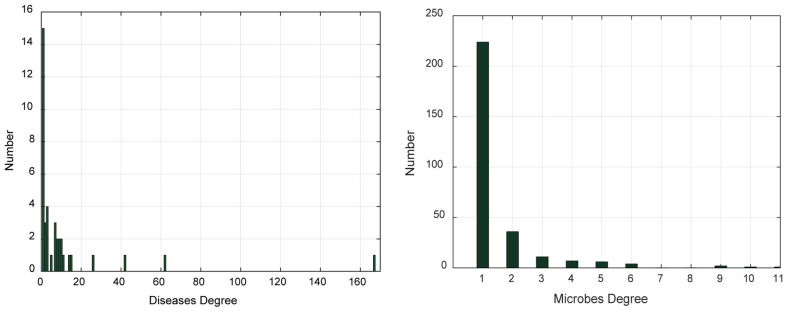
The degree distribution of diseases and microbes.

In addition, we analyzed the degree distribution characteristics of the microbe–disease association network (Table 1 and Figure 1). The degree of a disease represents the number of microbes related to this disease. The degree of a microbe represents the number of diseases related to this microbe. In the left graph of Figure 1, the abscissa indicates the range of disease degree, which presents how many microbes are related to each disease; the ordinate counts the number of each disease degree. In the right graph of Figure 1, the abscissa indicates the range of microbe degree, which shows how many diseases are related to each microbe; the ordinate counts the number of each microbe degree. On average, each disease is related to 11.54 microbes and each microbe is involved with 1.54 diseases.

**TABLE 1:** Characteristics of the microbe–disease association network.

**No. of microbes**	**No. of diseases**	**No. of microbe–disease associations**	**Avg. degree of diseases**	**Avg. degree of microbes**
292	39	450	11.54	1.54

### Gaussian Interaction Profile Kernel Similarity for Diseases and Microbes

According to the hypothesis that diseases have similar patterns with functionally similar microbes (Chen et al., 2016), we constructed a Gaussian interaction profile kernel similarity network for microbes and diseases based on the adjacency matrix *MD*, respectively. First, a binary vector *GIP*(*m*(*i*)) represents the interaction profiles of microbe *m*(*i*) by observing whether microbe *m*(*i*) has a known association with each disease or not (i.e., the *ith* row of adjacency matrix *MD*). Second, the Gaussian interaction profile kernel similarity between microbe *m*(*i*) and microbe *m*(*j*) could be defined as follows:

(1) $$K\,M\,\left(m\,\left(i\right),m\,\left(j\right)\right)=\exp\left(-\lambda_{m}\,{||G\,I\,P\,\left(m\,\left(i\right)\right)-G\,I\,P\,\left(m\,\left(j\right)\right)||}^{2}\right)$$

(2) $$\lambda_{m}=\lambda_{m}^{\prime }/\left(\frac{1}{n\,m}\,\sum_{i=1}^{n\,m}{||G\,I\,P\,\left(m\,\left(i\right)\right)||}^{2}\right)\,$$

where the parameter λ*_*m*_* is a regulation parameter, which could be obtained by normalizing a new parameter $\lambda_{m}^{\prime }$ to control the kernel bandwidth. For the sake of simplicity, we set $\lambda_{m}^{\prime }$ to 1 according to previous studies (van Laarhoven et al., 2011; Chen and Yan, 2013).

With the same processing, the Gaussian interaction profile kernel similarity between disease *d*(*i*) and disease *d*(*j*) was calculated as follows:

(3) $$K\,D\,\left(d\,\left(i\right),d\,\left(j\right)\right)=\exp\left(-\lambda_{d}\,{||G\,I\,P\,\left(d\,\left(i\right)\right)-G\,I\,P\,\left(d\,\left(j\right)\right)||}^{2}\right)$$

(4) $$\lambda_{d}=\lambda_{d}^{\prime }/\left(\frac{1}{n\,d}\,\sum_{i=1}^{n\,d}{||G\,I\,P\,\left(d\,\left(i\right)\right)||}^{2}\right)$$

where *GIP*(*d*(*i*)) represents the interaction profile of disease *d*(*i*) (i.e., the *ith* column of adjacency matrix *MD*). Here, the meaning of parameter λ*_*d*_* is the same as λ*_*m*_* and we also set the value of parameter $\lambda_{d}^{\prime }$ to 1 (van Laarhoven et al., 2011; Chen and Yan, 2013).

In the end, we could obtain the microbe Gaussian interaction profile kernel similarity matrix *KM* (*nm* × *nm*) and the disease Gaussian interaction profile kernel similarity matrix *KD*(*nd* × *nd*), respectively.

### Cosine Similarity for Diseases and Microbes

The calculation of disease cosine similarity is based on the assumption that if disease *d*(*i*) and disease *d*(*j*) are similar to each other (Xie et al., 2019), then, in the microbe–disease association matrix, pattern *MD*(:, *i*) (i.e., the *ith* column of the adjacency matrix *MD*) and pattern *MD*(:, *j*) (i.e., the *jth* column of adjacency matrix *MD*) should be similar to each other. The same assumption should also be true for microbes. Therefore, the cosine similarity between disease *d*(*i*) and disease *d*(*j*) is defined as follows:

(5) $$C\,D\,\left(d\,\left(i\right),d\,\left(j\right)\right)=\frac{M\,D\,\left(:,i\right)\cdot M\,D\,\left(:,j\right)}{||M\,D\,\left(:,i\right)||\times ||M\,D\,\left(:,j\right)||}$$

After calculating the disease–disease cosine similarity of each pair, the disease cosine similarity matrix *CD*(*nd* × *nd*) can be constructed.

Similarly, the cosine similarity between microbe *m*(*i*) and microbe *m*(*j*) is given:

(6) $$C\,M\,\left(m\,\left(i\right),m\,\left(j\right)\right)=\frac{M\,D\,\left(i,:\right)\cdot M\,D\,\left(j,:\right)}{||M\,D\,\left(i,:\right)||\times ||M\,D\,\left(j,:\right)||}$$

where *MD*(*i*,:) represents the *ith* row of adjacency matrix *MD*, and after calculating the microbe–microbe cosine similarity of each pair, the microbe cosine similarity matrix *CM*(*nm* × *nm*) can be constructed.

### Integrated Similarity for Diseases and Microbes

To make full use of disease Gaussian interaction profile kernel similarity matrix *KD* and disease cosine similarity matrix *CD*, a comprehensive disease similarity matrix *DS*(*nd* × *nd*) was constructed by integrating the *KD* and *CD* similarity matrices. We proposed a linear network fusion (LNF) method to integrate *KD* and *CD*, defined as follows:

(7) D⁢S⁢(d⁢(i),d⁢(j))=α⋅K⁢D⁢(d⁢(i),d⁢(j))+(1-α)⋅C⁢D⁢(d⁢(i),d⁢(j))

where entity *DS*(*d*(*i*), *d*(*j*)) represents the integrated similarity between disease *d*(*i*) and disease *d*(*j*) and α represents the weight of disease similarity matrix (0 < α < 1).

In the same way, microbe Gaussian interaction profile kernel similarity matrix *KM* and microbe cosine similarity matrix *CM* are integrated to a comprehensive microbe similarity matrix *MS*(*nm* × *nm*) as follows:

(8) M⁢S⁢(m⁢(i),m⁢(j))=β⋅K⁢M⁢(m⁢(i),m⁢(j))+(1-β)⋅C⁢M⁢(m⁢(i),m⁢(j))

where entity *MS*(*m*(*i*), *m*(*j*)) represents the integrated similarity between microbe *m*(*i*) and microbe *m*(*j*) and β represents the weight of microbe similarity matrix (0 < β < 1).

In the end, we obtained a comprehensive microbe similarity matrix *MS* and a comprehensive disease similarity matrix *DS*, respectively.

### HMDA-Pred

**FIGURE 2:**
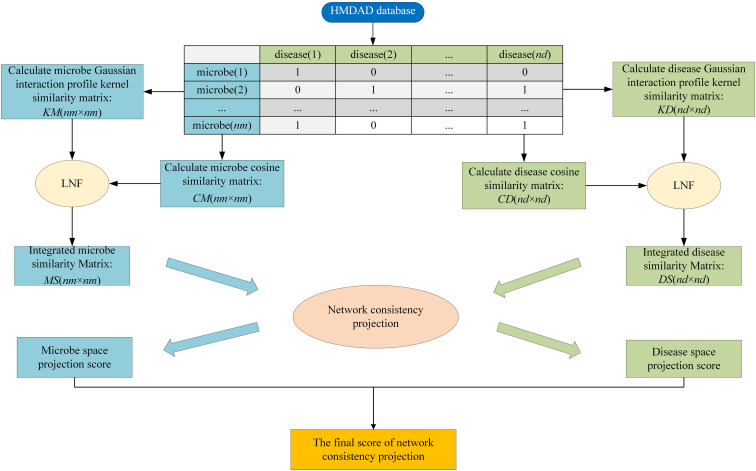
The flowchart of HMDA-Pred.

HMDA-Pred is a network-based computation approach to infer the disease-associated microbes based on the network consistency projection (*NCP*) algorithm. The flowchart of HMDA-Pred is shown in Figure 2. To begin with, based on known microbe–disease associations, we calculated the Gaussian interaction profile kernel similarity matrix and cosine similarity matrix for microbes and diseases, respectively. Then, we integrated two similarity matrices for microbes and for diseases through LNF, respectively. Finally, we uncovered the microbe–disease associations by scores obtained from the network consistency projection algorithm. The *NCP* algorithm has been successfully used to measure the similarity between nodes in the link prediction problems in a heterogeneous network (Gu et al., 2016; Bao et al., 2017). The following is how the *NCP* algorithm works in HMDA-Pred.

First, we calculated the disease space projection score as follows:

(9) $$N\,C\,P\,D\,\left(i,j\right)=\frac{M\,D\,\left(i,:\right)\cdot D\,S\,\left(:,j\right)}{\left|M\,D\,\left(i,:\right)\right|}$$

where *MD*(*i*,:) is composed of the associations of microbe *m*(*i*) and all diseases (i.e., the *ith* row of adjacency matrix *MD*), *DS*(:, *j*) is composed of the similarities of disease *d*(*j*) and all diseases (i.e., the *ith* column of adjacency matrix *DS*), and | *MD*(*i*,:)| represents the norm of *MD*(*i*,:). *NCPD*(*i*, *j*) represents the projection score of microbe *m*(*i*) and disease *d*(*j*) from the projection space of disease.

Second, we calculated the microbe space projection score as follows:

(10) $$N\,C\,P\,M\,\left(i,j\right)=\frac{M\,S\,\left(i,:\right)\cdot M\,D\,\left(:,j\right)}{\left|M\,D\,\left(:,j\right)\right|}$$

where *MD*(:, *j*) is composed of the associations of disease *d*(*i*) and all microbes (i.e., the ith column of adjacency matrix *MD*), *MS*(*i*,:) is composed of the similarities of microbe *m*(*i*) and all microbes (i.e., the *ith* row of adjacency matrix *DS*), and | *MD*(:, *j*)| represents the norm of *MD*(:, *j*). *NCPM*(*i*, *j*) represents the projection score of microbe *m*(*i*) and disease *d*(*j*) from the projection space of microbe.

Finally, we combined and normalized *NCPD* and *NCPM* as follows:

(11) $$N\,C\,P\,\left(i,j\right)=\frac{N\,C\,P\,D\,\left(i,j\right)+N\,C\,P\,M\,\left(i,j\right)}{\left|D\,S\,\left(:,j\right)\right|+\left|M\,S\,\left(i,:\right)\right|}$$

*NCP* is the final probability matrix of microbe–disease associations, and the element *NCP*(*i*, *j*) represents the final association score of network consistency projection of microbe *m*(*i*) and disease *d*(*j*).

## Results

### Performance Evaluation

To make the evaluation criteria consistent with existing methods, we performed LOOCV and 5-fold CV on our benchmark dataset, which are widely used not only in machine learning classification tasks based on sequence feature analysis but also in biological association prediction problems (Chen et al., 2016; Wang et al., 2017; Liu, 2019; Liu et al., 2019). For LOOCV, one of the 450 confirmed microbe–disease associations pairs was used as a test sample while the left 449 associations were used as the training samples. For 5-fold CV, we randomly divided the 450 confirmed microbe–disease association pairs into five subsets, where one subset is used as test samples and the remaining four subsets as training samples. The 5-fold CV was repeated 100 times to decrease the bias brought by the random splitting.

To visualize the performance of HMDA-Pred, the receiver operating characteristic (ROC) curve was used to plot the relationship between false-positive rate (1-specificity, 1-*Spe*) and true positive rate (sensitivity, *Sen*). The area under the ROC curve (AUC) was calculated, whose value of 1 represents perfect prediction performance, while 0.5 indicates purely random prediction performance (Chen et al., 2012, 2016; Fan and Shen, 2014; Pan and Shen, 2018). Moreover, we used the area under the precision-recall (PR) curve (AUPR) as an another indicator for model evaluation (Pan and Shen, 2019, 2020). In addition, we adopted accuracy (*Acc*), precision (*Pre*), Matthews’s correlation coefficient (*MCC*), and F1 score (*F*1) to further evaluate the model. They are defined as follows:

(12) $$S\,p\,e=\frac{T\,N}{T\,N+F\,P}$$

(13) $$S\,e\,n=\frac{T\,P}{T\,P+F\,N}$$

(14) $$A\,c\,c=\frac{T\,P+T\,N}{T\,P+T\,N+F\,N+F\,P}$$

(15) $$P\,r\,e=\frac{T\,P}{T\,P+F\,P}$$

(16) $$M\,C\,C=\frac{T\,P\times T\,N-F\,P\times F\,N}{\sqrt{\left(T\,P+F\,N\right)\times \left(T\,P+F\,P\right)\times \left(T\,N+F\,N\right)\times \left(T\,N+F\,P\right)}}$$

(17) $$F\,1=\frac{2\times T\,P}{2\times T\,P+F\,N+F\,P}$$

where *TP* represents the number of known microbe–disease associations that are correctly identified, *FP* represents the number of unknown microbe–disease associations that are incorrectly identified, *TN* represents the number of unknown microbe–disease associations that are correctly identified, and *FN* represents the number of known microbe–disease associations that are incorrectly identified.

### Parameter Selection

In this study, the parameters to be adjusted are α and β in LNF. We set the values of α and β from 0.1 to 0.9 with a step size of 0.1. In order to determine the best parameters, we ran LOOCV on the benchmark dataset to select the parameters with the best performance. As shown in Table 2, we observed that HMDA-Pred achieves the best AUC when α is 0.3 and β is 0.6.

**TABLE 2:** The AUC values of LNF integration method with different values of α and β.

	**β = 0.1**	**β = 0.2**	**β = 0.3**	**β = 0.4**	**β = 0.5**	**β = 0.6**	**β = 0.7**	**β = 0.8**	**β = 0.9**
α = 0.1	0.9505	0.9541	0.9551	0.9557	0.9563	0.9564	0.9564	0.9560	0.9551
α = 0.2	0.9522	0.9559	0.9574	0.9579	0.9581	0.9583	0.9582	0.9578	0.9568
α = 0.3	0.9527	0.9562	0.9576	0.9584	0.9588	**0.9589**	0.9586	0.9578	0.9566
α = 0.4	0.9524	0.9558	0.9573	0.9581	0.9586	0.9586	0.9582	0.9571	0.9556
α = 0.5	0.9518	0.9550	0.9564	0.9576	0.9581	0.9575	0.9571	0.9561	0.9541
α = 0.6	0.9510	0.9542	0.9556	0.9568	0.9571	0.9561	0.9556	0.9544	0.9523
α = 0.7	0.9503	0.9535	0.9547	0.9556	0.9554	0.9547	0.9539	0.9524	0.9499
α = 0.8	0.9491	0.9524	0.9536	0.9541	0.9540	0.9530	0.9519	0.9501	0.9469
α = 0.9	0.9476	0.9510	0.9521	0.9526	0.9523	0.9512	0.9499	0.9477	0.9436

*The bold value is the highest AUC value.*

### Comparison With Other Integration Strategies

**FIGURE 3:**
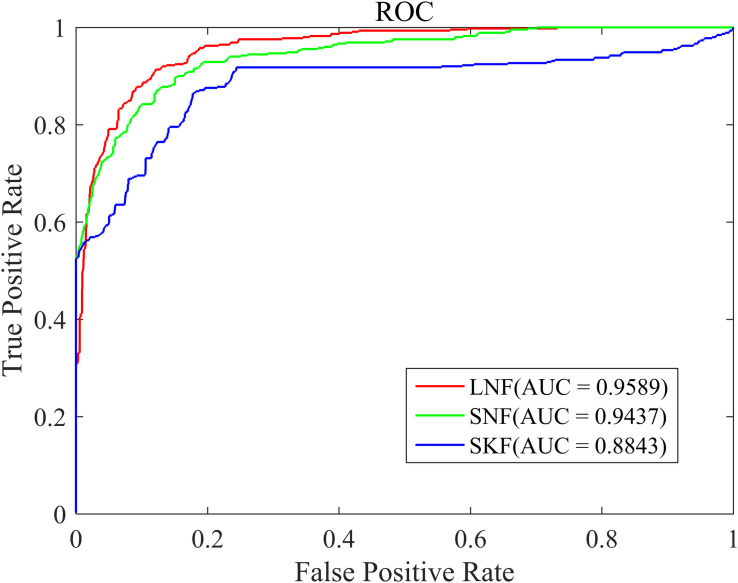
The ROC curve of three integration strategies.

The similarity integration strategy proposed in this study is a linear network fusion (LNF) method. In order to verify the superior integration performance of the LNF, we compared LNF with two common similarity fusion strategies: similarity network fusion (SNF) (Zheng et al., 2017) and similarity kernel fusion (SKF) (Jiang et al., 2018; Xie et al., 2019). As shown in Figure 3, based on the LOOCV scheme, we plotted the ROC curve of three different integration methods. The AUC value of LNF achieved 0.9589, while those of SNF and SKF were 0.9437 and 0.8843, respectively. It can be seen that the AUC value of LNF is higher than that of SNF and SKF. Therefore, in the HMDA-Pred method, the performance of LNF is superior to the other two fusion methods in terms of the prediction accuracy of the microbe–disease associations.

### Comparison With Single Similarity

In this study, we proposed to integrate different similarity data of microbes (i.e., Gaussian interaction profile kernel similarity and cosine similarity for microbes) and different similarity data of diseases (i.e., Gaussian interaction profile kernel similarity and cosine similarity for diseases) by LNF, respectively. The integration effect was verified by designing comparative experiments, including all combinations of single similarity data of diseases and microbes. The experimental results are shown in Table 3. The proposed strategy of using LNF to integrate Gaussian interaction profile kernel similarity data and cosine similarity data presented the highest AUC values in LOOCV and 5-fold CV, which were 0.9589 and 0.9361 ± 0.0037, respectively.

**TABLE 3:** The AUC values of HMDA-Pred and other single similarity in LOOCV and 5-fold CV.

**Microbe similarity**	**Disease similarity**	**LOOCV**	**5-fold CV**
Gaussian	Gaussian	0.9295	0.9053 ± 0.0035
Gaussian	Cosine	0.9480	0.9154 ± 0.0044
Cosine	Cosine	0.9333	0.9049 ± 0.0062
Cosine	Gaussian	0.9287	0.9033 ± 0.0080
Gaussian + cosine	Gaussian + cosine	**0.9589**	**0.9361 ± 0.0037**

*The bold values are the highest AUC value in LOOCV and 5-fold CV respectively.*

### Comparison With Other Existing Methods

**FIGURE 4:**
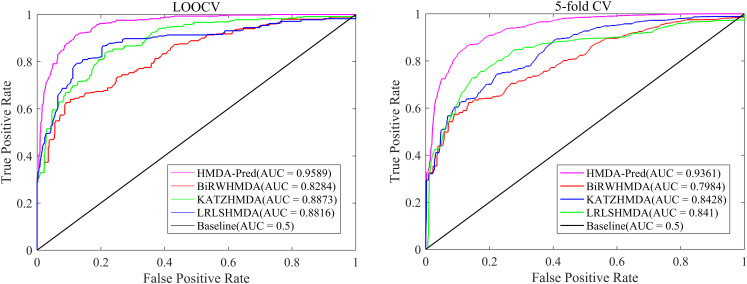
The ROC curve of different methods in LOOCV and 5-fold CV.

In order to further verify the superior predictive performance of HMDA-Pred, we compared HMDA-Pred with three state-of-the-art methods used to predict microbe–disease associations, namely, KATZHMDA (Chen et al., 2016), BiRWHMDA (Zou et al., 2017), and LRLSHMDA (Wang et al., 2017). Figure 4 shows the comparisons of the AUC values between different methods based on the benchmark data set. By LOOCV, the AUC values of KATZHMDA, BiRWHMDA, LRLSHMDA, and HMDA-Pred are 0.8873, 0.8284, 0.8816, and 0.9589, respectively. However, after repeating for 100 times the 5-fold CV, the AUC values of KATZHMDA, BiRWHMDA, LRLSHMDA, and HMDA-Pred are 0.8428 ± 0.0035, 0.7984 ± 0.0027, 0.8410 ± 0.0052, and 0.9361 ± 0.0037, respectively.

**FIGURE 5:**
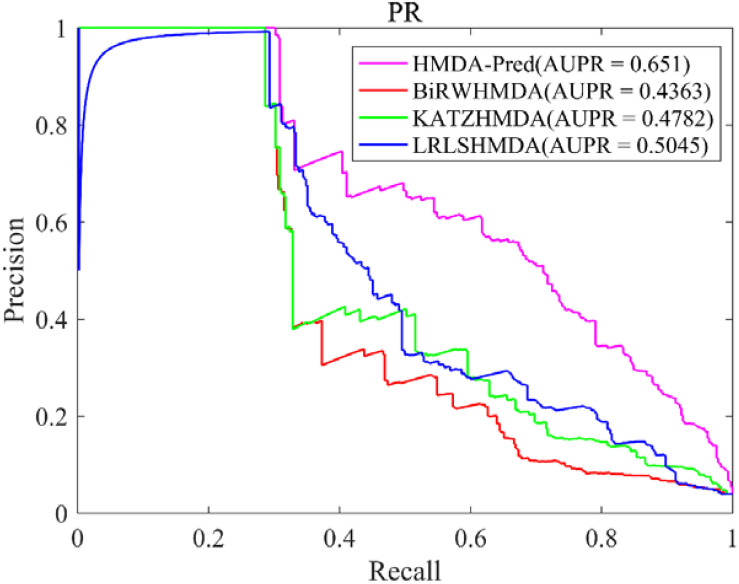
The PR curve of different methods in LOOCV.

In this study, the known microbe–disease associations are far less than unknown microbe–disease associations in the benchmark dataset, which is imbalanced. Therefore, the AUPR value (area under the PR curve) is an indispensable model evaluation indicator to show the balance of recall and precision, which is suitable to investigate the performance of different methods in the imbalanced dataset (Li et al., 2018). Based on the benchmark data set, we plotted the PR curve of each method and calculated the AUPR value of each method by LOOCV. As shown in Figure 5, the AUPR values of HMDA-Pred, BiRWHMDA, KATZHMAD, and LRLSHMDA are 0.6510, 0.4363, 0.4782, and 0.5045, respectively, which reflects that the performance of HMDA-Pred is better than the other three methods in the case of imbalanced data set.

Moreover, we used two stringency levels to further measure the predictive performance of the model (Sun et al., 2016). As shown in Table 4, at the medium specificity level (*Spe* = 95.0%), the *Sen*, *Acc*, *Pre*, *F*1, and *MCC* of HMDA-Pred are 79.1, 94.4, 39.4, 52.6, and 53.4%, respectively; of KATZHMDA are 59.7, 93.6, 32.9, 42.4, and 41.2%, respectively; that of LRLSHMDA are 55.1, 93.4, 31.2, 39.8, and 38.3%, respectively; and that of BiRWHMDA are 46.9, 93.1, 27.8, 34.9, and 32.7%, respectively. When *Spe* = 99.0% (i.e., at the high specificity level), the *Sen*, *Acc*, *Pre*, *F1*, and *MCC* of HMDA-Pred are 49.9, 97.1, 67.3, 57.2, and 56.4%, which are higher than those of KATZHMDA, LRLSHMDA, and BiRWHMDA methods.

**TABLE 4:** The evaluation indicators of different methods at two stringency levels.

	**HMDA-Pred (%)**	**KATZHMDA (%)**	**LRLSHMDA (%)**	**BiRWHMDA (%)**
***Spe* = 99.0%**				
*Sen*	49.9	32.9	37.8	32.9
*Acc*	97.1	96.4	96.6	96.4
*Pre*	67.3	57.6	60.9	57.6
*F*1	57.2	41.9	46.6	41.9
*MCC*	56.4	41.8	46.4	41.8
***Spe* = 95.0%**				
*Sen*	79.1	59.7	55.1	46.9
*Acc*	94.4	93.6	93.4	93.1
*Pre*	39.4	32.9	31.2	27.8
*F*1	52.6	42.4	39.8	34.9
*MCC*	53.4	41.2	38.3	32.7
				

**FIGURE 6:**
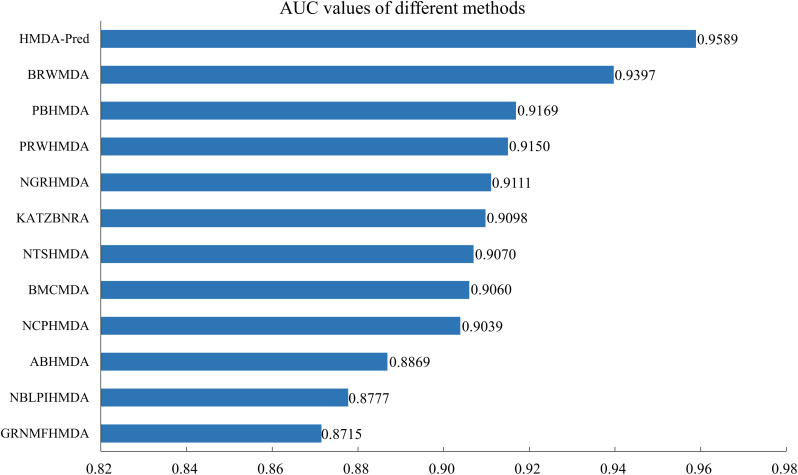
AUC values of different methods in LOOCV.

In addition, we compared the HMDA-Pred method with the BRWMDA (Yan et al., 2019), PBHMDA (Huang et al., 2017), PRWHMDA (Wu et al., 2018), NGRHMDA (Huang et al., 2017), KATZBNRA (Li et al., 2019), NTSHMDA (Luo and Long, 2018), BMCMDA (Shi et al., 2018), NCPHMDA (Bao et al., 2017), ABHMDA (Peng et al., 2018), NBLPIHMDA (Wang et al., 2019), and GRNMFHMDA (He et al., 2018) methods. As shown in Figure 6, these AUC values extracted from the original papers include 0.9397, 0.9169, 0.9150, 0.9111, 0.9098, 0.9070, 0.9060, 0.9039, 0.8869, 0.8777, and 0.8715. The AUC value of HMDA-Pred is 0.9589, which is higher than those of other 10 methods by 0.0192, 0.0420, 0.0439, 0.0478, 0.0491, 0.0519, 0.0529, 0.0550, 0.0720, 0.0812, and 0.0874, respectively. The above experimental results fully demonstrate that the HMDA-Pred method has better prediction performance than the other state-of-the-art methods.

### Case Studies

In this section, we investigated the top 10 microbes predicted by HMDA-Pred to be potentially associated with asthma, colon cancer, and inflammatory bowel disease, respectively. Then, we validated the predicted results by searching the relevant literatures, with the purpose of further evaluating the performance of HMDA-Pred.

Asthma is a common chronic disease, generally considered to be caused by a combination of genetic and environmental factors (Althani et al., 2016). The top 10 microbes predicted by the HMDA-Pred method have been confirmed to be potentially related to asthma in the relevant literatures, as shown in Table 5. Colon cancer is a common gastrointestinal malignant tumor with high morbidity and mortality (Bao et al., 2017). We selected the top 10 microbes that were potentially related to colon cancer predicted by HMDA-Pred, and through searching the relevant literatures, we confirmed that 8 of them were related to colon cancer, as shown in Table 6. Inflammatory bowel disease is also known as non-specific enteritis or idiopathic enteritis, whose etiology has not been completely clear. Also, there is no cure for it in medicine currently (Wu et al., 2018). The top 10 microbes most likely to be associated with inflammatory bowel disease were predicted by HMDA_Pred, which was confirmed by relevant literatures, as shown in Table 7.

**TABLE 5:** The top 10 potential asthma-related microbes predicted by HMDA-Pred.

**Rank**	**Microbe**	**Evidence**
1	Firmicutes	PMID:23265859
2	Pseudomonas	PMID:13268970
3	Clostridium coccoides	PMID:21477358
4	Actinobacteria	PMID:28947029
5	Burkholderia	PMID:24451910
6	Lactobacillus	PMID:20592920
7	Lachnospiraceae	Ciaccio et al., 2014
8	Propionibacterium	PMID:27433177
9	Propionibacterium acnes	PMID:27433177
10	Fusobacterium nucleatum	Dang et al., 2013

**TABLE 6:** The top 10 potential colon cancer-related microbes predicted by HMDA-pred.

**Rank**	**Microbe**	**Evidence**
1	Proteobacteria	PMID:24603888
2	Clostridium coccoides	PMID:19807912
3	Haemophilus	PMID:22761885
4	Lactobacillus	PMID:15828052
5	Staphylococcus	Unconfirmed
6	Helicobacter pylori	PMID:11774957
7	Lachnospiraceae	PMID:21850056
8	Actinobacteria	PMID:24316595
9	Faecalibacterium prausnitzii	Unconfirmed
10	Streptococcus	PMID:21247505
		

**TABLE 7:** The top 10 potential inflammatory bowel disease-related microbes predicted by HMDA-Pred.

**Rank**	**Microbe**	**Evidence**
1	Clostridium coccoides	PMID:19235886
2	Bacteroidetes	PMID:25307765
3	Staphylococcus	Luo and Long, 2018
4	Firmicutes	PMID:25307765
5	Prevotella	PMID:25307765
6	Helicobacter pylori	PMID:22221289
7	Clostridium difficile	Luo and Long, 2018
8	Haemophilus	PMID:24013298
9	Propionibacterium	PMID:26640113
10	Propionibacterium acnes	PMID:26640113

## Discussion

Effective computational methods can predict microbe–disease associations in a more efficient and low-cost manner, thus becoming an important aid to biological experimental methods.

In this study, we present a novel prediction method called HMDA-Pred based on known microbe–disease associations, Gaussian interaction profile kernel similarity for microbes and diseases, and cosine similarity for microbes and diseases to infer disease-associated microbes. HMDA-Pred achieved AUC values of 0.9589 and 0.9361 ± 0.0037 in the LOOCV and 5-fold CV, respectively. In addition, we conducted case studies of asthma, colon cancer, and inflammatory bowel disease to further validate the predictive performance of HMDA-Pred, where 10, 8, and 10 of the top 10 candidate microbes were confirmed from literatures, respectively. Given the superior performance of HMDA-Pred, we expect HMDA-Pred to be a promising and effective tool for assisting clinical and biological research.

There are several reasons why HMDA-Pred performs well in microbe–disease associations prediction. First, the datasets used in HMDA-Pred are relatively more reliable. Secondly, a linear network fusion method is used to fuse multiple similarity networks to obtain an informative matrix. Third, network consistency projection executed on microbe and disease spatial networks is efficient and reliable. There is also room for improvement of HMDA-Pred in future work. First, although the predictive performance of HMDA-Pred has improved compared to previous methods, it will be further improved if more reliable similarities are considered, such as the semantic similarity of diseases and the functional similarity of microbes. Second, HMDA-Pred will inevitably lead to a bias in disease with more known related microbes due to data imbalance.

## Data Availability Statement

All datasets and code link for this study are included in the article.

## Author Contributions

QZ developed the prediction model and designed the experiments. QZ, YF, and MC analyzed the experiment and results and wrote the manuscript. MC and WW proofread the manuscript. All authors contributed to the article and approved the submitted version.

## Conflict of Interest

The authors declare that the research was conducted in the absence of any commercial or financial relationships that could be construed as a potential conflict of interest.

## Footnotes

1 www.cuilab.cn//hmdad

## References

[B1] Althani A. A. Marei H. E. Hamdi W. S. Nasrallah G. K. El Zowalaty M. E. Al Khodor S. (2016). Human microbiome and its association with health and diseases. *J. Cell. Physiol.* 231 1688–1694. 10.1002/jcp.25284 26660761

[B2] Bao W. Jiang Z. Huang D.-S. (2017). Novel human microbe-disease association prediction using network consistency projection. *BMC Bioinf.* 18:543. 10.1186/s12859-017-1968-2 29297304 PMC5751545

[B3] Chen X. Huang Y.-A. You Z.-H. Yan G.-Y. Wang X.-S. (2016). A novel approach based on KATZ measure to predict associations of human microbiota with non-infectious diseases. *Bioinformatics* 33 733–739. 10.1093/bioinformatics/btw715 28025197

[B4] Chen X. Liu M. Yan G. (2012). RWRMDA: predicting novel human microRNA-disease associations. *Mol Biosyst.* 8 2792–2798. 10.1039/c2mb25180a 22875290

[B5] Chen X. Yan G.-Y. (2013). Novel human lncRNA-disease association inference based on lncRNA expression profiles. *Bioinformatics* 29 2617–2624. 10.1093/bioinformatics/btt426 24002109

[B6] Ciaccio C. E. Kennedy K. Barnes C. S. Portnoy J. M. Rosenwasser L. J. (2014). The home microbiome and childhood asthma. *J. Allergy Clin. Immunol.* 133:AB70 10.1016/j.jaci.2013.12.274

[B7] Dang H. T. Park H. K. Shin J. W. Park S.-G. Kim W. (2013). Analysis of oropharyngeal microbiota between the patients with bronchial asthma and the non-asthmatic persons. *J. Bacteriol. Virol.* 43 270–278. 10.4167/jbv.2013.43.4.270 27650591

[B8] Fan Y.-X. Shen H.-B. (2014). Predicting pupylation sites in prokaryotic proteins using pseudo-amino acid composition and extreme learning machine. *Neurocomputing* 128 267–272. 10.1016/j.neucom.2012.11.058

[B9] Frank D. N. Amand A. L. S. Feldman R. A. Boedeker E. C. Harpaz N. Pace N. R. (2007). Molecular-phylogenetic characterization of microbial community imbalances in human inflammatory bowel diseases. *Proc. Natl. Acad. Sci. U.S.A.* 104 13780–13785. 10.1073/pnas.0706625104 17699621 PMC1959459

[B10] Fredricks D. N. Fiedler T. L. Marrazzo J. M. (2005). Molecular identification of bacteria associated with bacterial vaginosis. *N. Engl. J. Med.* 353 1899–1911. 10.1056/NEJMoa043802 16267321

[B11] Gu C. Liao B. Li X. Li K. (2016). Network consistency projection for human miRNA-disease associations inference. *Sci. Rep.* 6:36054. 10.1038/srep36054 27779232 PMC5078764

[B12] He B.-S. Peng L.-H. Li Z. (2018). Human microbe-disease association prediction with graph regularized non-negative matrix factorization. *Front. Microbiol.* 9:2560. 10.3389/fmicb.2018.02560 30443240 PMC6223245

[B13] Hilty M. Burke C. Pedro H. Cardenas P. Bush A. Bossley C. (2010). Disordered microbial communities in asthmatic airways. *PLoS ONE* 5:e8578. 10.1371/journal.pone.0008578 20052417 PMC2798952

[B14] Hoppe B. Groothoff J. W. Hulton S.-A. Cochat P. Niaudet P. Kemper M. J. (2011). Efficacy and safety of oxalobacter formigenes to reduce urinary oxalate in primary hyperoxaluria. *Nephrol. Dial. Transplant.* 26 3609–3615. 10.1093/ndt/gfr107 21460356

[B15] Huang Y.-A. You Z.-H. Chen X. Huang Z.-A. Zhang S. Yan G.-Y. (2017). Prediction of microbe-disease association from the integration of neighbor and graph with collaborative recommendation model. *J. Transl. Med.* 15:209. 10.1186/s12967-017-1304-7 29037244 PMC5644104

[B16] Huang Z.-A. Chen X. Zhu Z. Liu H. Yan G.-Y. You Z.-H. (2017). PBHMDA: path-based human microbe-disease association prediction. *Front. Microbiol.* 8:233. 10.3389/fmicb.2017.00233 28275370 PMC5319991

[B17] Jiang L. Ding Y. Tang J. Guo F. (2018). MDA-SKF: similarity kernel fusion for accurately discovering miRNA-disease association. *Front. Genet.* 9:618. 10.3389/fgene.2018.00618 30619454 PMC6295467

[B18] Kostic A. D. Gevers D. Pedamallu C. S. Michaud M. Duke F. Earl A. M. (2012). Genomic analysis identifies association of *Fusobacterium* with colorectal carcinoma. *Genome Res.* 22 292–298. 10.1101/gr.126573.111 22009990 PMC3266036

[B19] Kouzuma A. Kato S. Watanabe K. (2015). Microbial interspecies interactions: recent findings in syntrophic consortia. *Front. Microbiol.* 6:477. 10.3389/fmicb.2015.00477 26029201 PMC4429618

[B20] Li G. Luo J. Xiao Q. Liang C. Ding P. (2018). Predicting microRNA-disease associations using label propagation based on linear neighborhood similarity. *J. Biomed. Inf.* 82 169–177. 10.1016/j.jbi.2018.05.005 29763707

[B21] Li S. Xie M. Liu X. (2019). A novel approach based on bipartite network recommendation and KATZ model to predict potential micro-disease associations. *Front. Genet.* 10:1147. 10.3389/fgene.2019.01147 31803235 PMC6873782

[B22] Liu B. (2019). BioSeq-analysis: a platform for DNA, RNA and protein sequence analysis based on machine learning approaches. *Brief Bioinform.* 20 1280–1294. 10.1093/bib/bbx165 29272359

[B23] Liu B. Gao X. Zhang H. (2019). BioSeq-Analysis2.0: an updated platform for analyzing DNA, RNA and protein sequences at sequence level and residue level based on machine learning approaches. *Nucleic Acids Res.* 47:e127. 10.1093/nar/gkz740 31504851 PMC6847461

[B24] Luo J. Long Y. (2018). NTSHMDA: prediction of human microbe-disease association based on random walk by integrating network topological similarity. *IEEE/ACM Trans. Comput. Biol. Bioinf.* 10.1109/tcbb.2018.2883041∗ 30489271

[B25] Ma W. Zhang L. Zeng P. Huang C. Li J. Geng B. (2016). An analysis of human microbe-disease associations. *Briefings Bioinf.* 18 85–97. 10.1093/bib/bbw005 26883326

[B26] Methé B. A. Nelson K. E. Pop M. Creasy H. H. Giglio M. G. Huttenhower C. (2012). A framework for human microbiome research. *Nature* 486:215. 10.1038/nature11209 22699610 PMC3377744

[B27] Pan X. Shen H. B. (2018). Predicting RNA-protein binding sites and motifs through combining local and global deep convolutional neural networks. *Bioinformatics* 34 3427–3436. 10.1093/bioinformatics/bty364 29722865

[B28] Pan X. Shen H.-B. (2019). Inferring disease-associated microRNAs using semi-supervised multi-label graph convolutional networks. *iScience* 20 265–277. 10.1016/j.isci.2019.09.013 31605942 PMC6817654

[B29] Pan X. Shen H.-B. (2020). Scoring disease-microRNA associations by integrating disease hierarchy into graph convolutional networks. *Pattern Recognit.* 105:107385 10.1016/j.patcog.2020.107385

[B30] Peng L.-H. Yin J. Zhou L. Liu M.-X. Zhao Y. (2018). Human microbe-disease association prediction based on adaptive boosting. *Front. Microbiol.* 9:2440. 10.3389/fmicb.2018.02440 30356751 PMC6189371

[B31] Qin J. Li R. Raes J. Arumugam M. Burgdorf K. S. Manichanh C. (2010). A human gut microbial gene catalogue established by metagenomic sequencing. *Nature* 464 59–65. 10.1038/nature08821 20203603 PMC3779803

[B32] Sears C. L. Garrett W. S. (2014). Microbes, microbiota, and colon cancer. *Cell Host Microbe* 15 317–328. 10.1016/j.chom.2014.02.007 24629338 PMC4003880

[B33] Shi J. Huang H. Zhang Y. Cao J. Yiu S. (2018). BMCMDA: a novel model for predicting human microbe-disease associations via binary matrix completion. *BMC Bioinformatics* 19:281. 10.1186/s12859-018-2274-3 30367598 PMC6101089

[B34] Sobhani I. Tap J. Roudot-Thoraval F. Roperch J. P. Letulle S. Langella P. (2011). Microbial dysbiosis in colorectal cancer (CRC) patients. *PLoS ONE* 6:e16393. 10.1371/journal.pone.0016393 21297998 PMC3029306

[B35] Sommer F. Bäckhed F. (2013). The gut microbiota-masters of host development and physiology. *Nat. Rev. Microbiol.* 11:227. 10.1038/nrmicro2974 23435359

[B36] Sun D. Li A. Feng H. Wang M. (2016). NTSMDA: prediction of miRNA-disease associations by integrating network topological similarity. *Mol. BioSyst.* 12 2224–2232. 10.1039/c6mb00049e 27153230

[B37] Turnbaugh P. J. Hamady M. Yatsunenko T. Cantarel B. L. Duncan A. Ley R. E. (2009). A core gut microbiome in obese and lean twins. *Nature* 457:480. 10.1038/nature07540 19043404 PMC2677729

[B38] van Laarhoven T. Nabuurs S. B. Marchiori E. (2011). Gaussian interaction profile kernels for predicting drug-target interaction. *Bioinformatics* 27:3036–3043. 10.1093/bioinformatics/btr500 21893517

[B39] Ventura M. O’flaherty S. Claesson M. J. Turroni F. Klaenhammer T. R. Van Sinderen D. (2009). Genome-scale analyses of health-promoting bacteria: probiogenomics. *Nat. Rev. Microbiol.* 7 61–71. 10.1038/nrmicro2047 19029955

[B40] Wang F. Huang Z.-A. Chen X. Zhu Z. Wen Z. Zhao J. (2017). LRLSHMDA: laplacian regularized least squares for human microbe-disease association prediction. *Sci. Rep.* 7:7601. 10.1038/s41598-017-08127-2 28790448 PMC5548838

[B41] Wang L. Wang Y. Li H. Feng X. Yuan D. Yang J. (2019). A bidirectional label propagation based computational model for potential microbe-disease association prediction. *Front. Microbiol.* 10:684. 10.3389/fmicb.2019.00684 31024481 PMC6465563

[B42] Wu C. Gao R. Zhang D. Han S. Zhang Y. (2018). PRWHMDA: human microbe-disease association prediction by random walk on the heterogeneous network with PSO. *Int. J. Biol. Sci.* 14 849–857. 10.7150/ijbs.24539 29989079 PMC6036753

[B43] Xie G. Meng T. Luo Y. Liu Z. (2019). SKF-LDA: similarity kernel fusion for predicting lncRNA-disease association. *Mol. Ther. Nucleic Acids* 18 45–55. 10.1016/j.omtn.2019.07.022 31514111 PMC6742806

[B44] Yan C. Duan G. Wu F. Pan Y. Wang J. (2019). BRWMDA:Predicting microbe-disease associations based on similarities and bi-random walk on disease and microbe networks. *IEEE/ACM Trans. Comput. Biol. Bioinf.* 10.1109/TCBB.2019.2907626∗ 30932846

[B45] Zheng X. Wang Y. Tian K. Zhou J. Guan J. Luo L. (2017). Fusing multiple protein-protein similarity networks to effectively predict lncRNA-protein interactions. *BMC Bioinf.* 18(Suppl. 12):420. 10.1186/s12859-017-1819-1 29072138 PMC5657051

[B46] Zou S. Zhang J. Zhang Z. (2017). A novel approach for predicting microbe-disease associations by bi-random walk on the heterogeneous network. *PLoS ONE* 12:e0184394. 10.1371/journal.pone.0184394 28880967 PMC5589230

